# Extra-Virgin Olive Oil Enhances the Blood–Brain Barrier Function in Mild Cognitive Impairment: A Randomized Controlled Trial

**DOI:** 10.3390/nu14235102

**Published:** 2022-12-01

**Authors:** Amal Kaddoumi, Thomas S. Denney, Gopikrishna Deshpande, Jennifer L. Robinson, Ronald J. Beyers, David T. Redden, Domenico Praticò, Tassos C. Kyriakides, Bonian Lu, Anna N. Kirby, Darren T. Beck, Nancy D. Merner

**Affiliations:** 1 Department of Drug Discovery and Development, Harrison College of Pharmacy, Auburn University, Auburn, AL 36849, USA; 2 Center for Neuroscience, Auburn University, Auburn, AL 36849, USA; 3 Auburn University MRI Research Center, Department of Electrical & Computer Engineering, Auburn University, Auburn, AL 36849, USA; 4 Department of Psychological Sciences, Auburn University, Auburn, AL 36849, USA; 5 Alabama Advanced Imaging Consortium, Birmingham, AL 36849, USA; 6 Key Laboratory for Learning and Cognition, School of Psychology, Capital Normal University, Beijing 100037, China; 7 Department of Psychiatry, National Institute of Mental Health and Neurosciences, Bangalore 560030, India; 8 Centre for Brain Research, Indian Institute of Science, Bangalore 560012, India; 9 Department of Heritage Science and Technology, Indian Institute of Technology, Hyderabad 502285, India; 10 Biomedical Affairs and Research, Edward Via College of Osteopathic Medicine, Auburn University, Auburn, AL 36849, USA; 11 Alzheimer’s Center at Temple, Lewis Katz School of Medicine Temple University, Philadelphia, PA 19140, USA; 12 Yale School of Public Health, New Haven, CT 06510, USA; 13 Department of Cell Biology and Physiology and Preventive Medicine and Public Health, Edward Via College of Osteopathic Medicine, Auburn University, Auburn, AL 36849, USA; 14 Department of Cell Biology and Physiology, Via College of Osteopathic Medicine, Auburn University, Auburn, AL 36849, USA; 15 Department of Pathobiology, College of Veterinary Medicine, Auburn University, Auburn, AL 36849, USA

**Keywords:** Alzheimer’s disease, mild cognitive impairment, blood–brain barrier, contrast-enhanced MRI, functional MRI, extra-virgin olive oil, refined olive oil, amyloid beta peptides, tau, cognitive function

## Abstract

Mild cognitive impairment (MCI) and early Alzheimer’s disease (AD) are characterized by blood–brain barrier (BBB) breakdown leading to abnormal BBB permeability ahead of brain atrophy or dementia. Previous findings in AD mouse models have reported the beneficial effect of extra-virgin olive oil (EVOO) against AD, which improved BBB and memory functions and reduced brain amyloid-β (Aβ) and related pathology. This work aimed to translate these preclinical findings to humans in individuals with MCI. We examined the effect of daily consumption of refined olive oil (ROO) and EVOO for 6 months in MCI subjects on BBB permeability (assessed by contrast-enhanced MRI), and brain function (assessed using functional-MRI) as the primary outcomes. Cognitive function and AD blood biomarkers were also assessed as the secondary outcomes. Twenty-six participants with MCI were randomized with 25 participants completed the study. EVOO significantly improved clinical dementia rating (CDR) and behavioral scores. EVOO also reduced BBB permeability and enhanced functional connectivity. While ROO consumption did not alter BBB permeability or brain connectivity, it improved CDR scores and increased functional brain activation to a memory task in cortical regions involved in perception and cognition. Moreover, EVOO and ROO significantly reduced blood Aβ_42_/Aβ_40_ and p-tau/t-tau ratios, suggesting that both altered the processing and clearance of Aβ. In conclusion, EVOO and ROO improved CDR and behavioral scores; only EVOO enhanced brain connectivity and reduced BBB permeability, suggesting EVOO biophenols contributed to such an effect. This proof-of-concept study justifies further clinical trials to assess olive oil’s protective effects against AD and its potential role in preventing MCI conversion to AD and related dementias.

## 1. Introduction

Significant evidence suggests that the neurodegenerative process of Alzheimer’s disease (AD) is associated with a dysfunctional blood–brain barrier (BBB), which could be caused by aging and the presence of vascular conditions such as hypertension, atherosclerosis, type II diabetes, hypercholesterolemia, and cardiovascular diseases [1,2,3,4,5,6]. The BBB functions to maintain brain homeostasis and normal neuronal function. The endothelial cells of the BBB have highly controlled permeability towards plasmatic compounds and ions and have high trans-endothelial electrical resistance [7]. Thus, any changes in the BBB function may allow toxic molecules access to the brain and impair the clearance of neurotoxic molecules, such as amyloid-β (Aβ) peptides and tau proteins, which could lead to oxidative stress, neuroinflammation, disruption of the functional brain network, and impairment in cerebral blood flow revealed in patients with mild cognitive impairment (MCI), a condition that could advance to AD and related dementias [8]. Such alterations in the BBB in MCI and AD point toward the BBB as a target for therapeutic interventions and suggest that identifying strategies to maintain and rectify BBB function, reduce the accumulation of brain Aβ and hyperphosphorylated tau, and maintain neuronal function is essential to prevent or delay the onset of AD.

Dietary lifestyle is a risk factor that, by modification, could reduce the risk or delay the onset of AD. Several epidemiological and clinical studies suggested that adherence to the Mediterranean diet improves cognitive function and slows AD progression [9,10,11]. Olive oil is one of the main elements of the Mediterranean diet. Extra-virgin olive oil (EVOO) is a high-quality olive oil obtained from the first pressing of olive fruit purely by mechanical means. EVOO composition is primarily the glycerol fraction (~95%, rich in monounsaturated fatty acids) and the nonglycerol fraction, which contains phenolic compounds (~5%) [12]. Recent studies have shown that the phenolic compounds in EVOO are antioxidant molecules that can scavenge the toxic effects of oxygen metabolism, such as free radical formation, thus protecting cells against oxidative damage and contributing significantly to human health [13,14,15]. Our studies demonstrated that the addition of EVOO with variable levels of phenolic content (low to high range) to the diet of AD mouse models restored the BBB function and reduced brain Aβ- and tau- related pathologies, oxidative stress, and neuroinflammation, which collectively was associated with improved memory function [16,17,18,19,20]. However, whether such promising preclinical findings translate to humans and whether the observed effect is due to EVOO-phenolic fraction remains to be evaluated. In this work, we hypothesized that olive oil, specifically EVOO, restores the BBB function, strengthens functional brain networks, improves cognitive function, and reduces AD pathological markers. To test this hypothesis, in this proof-of-concept study, we aimed to evaluate the effect of EVOO in MCI participants and compare its effect with refined olive oil (ROO; null in phenolic fraction) using a variety of metrics: (i) the BBB integrity obtained from contrast-enhanced MRI imaging, and brain function and network connectivity obtained by functional MRI (fMRI), (ii) cognitive function assessed using neuropsychological evaluation, and (iii) blood Aβ, tau, phospho-tau 181, and neurofilament light (NFL) levels.

## 2. Materials and Methods

### 2.1. Subjects and Study Design

**Figure 1:**
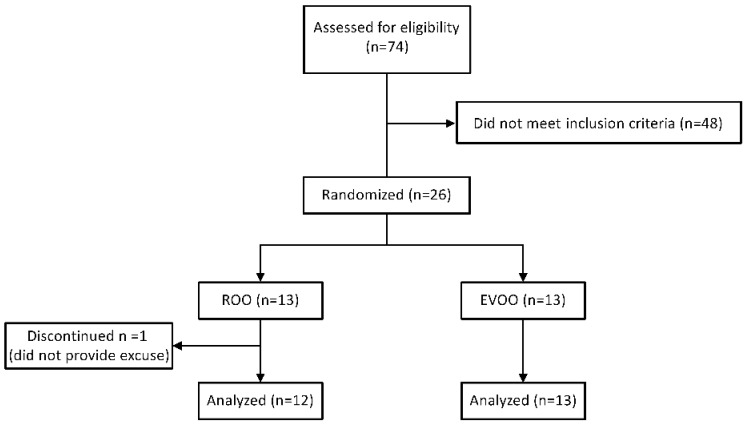
Trial profile.

Individuals with MCI were recruited from the community and assessed for eligibility at Auburn University MRI Research Center (AU-MRI-RC). The study and procedures were approved by the Institutional Review Board of Auburn University for compliance and ethical regulations. Informed consent forms were obtained from all participants before study enrollment. All participants underwent neuropsychological evaluations for inclusion, as described below. Eligible participants, directly after baseline measurements, were randomly assigned to either the EVOO or ROO group. For randomization, participants were requested to blindly withdraw a paper labeled with either EVOO or ROO. Study participants underwent neuropsychological evaluations, imaging, and blood samples collection twice, before (baseline) and at the end of the olive oil consumption for 6 months. The study profile is illustrated in Figure 1. ClinicalTrials.gov Identifier number is NCT03824197 (Auburn University Research on Olive Oil for Alzheimer’s Disease (AU-ROOAD)).

Twenty-six participants were enrolled in the study. One participant did not complete the post-intervention session and thus was excluded from the analysis. The withdrawn participant did not provide a reason for not continuing the study. Thirteen participants were enrolled in the EVOO group and 12 participants in the ROO group. The EVOO group received 30 mL daily of The Governor EVOO containing 1200 mg/kg of total polyphenols for 6 months. The Governor EVOO was selected based on our previous preclinical studies as it is rich in polyphenols [16,17]. The certificate of analysis of the total phenolic content of The Governor EVOO is illustrated in the Supplementary material demonstrating the majority of the phenolic content consists of oleocanthal (621 mg/kg) and oleacein (344 mg/kg). The ROO group received 30 mL daily of refined olive oil that contains a null level of polyphenols (Bertolli^®^ Extra Light Olive Oil) for 6 months. Every month, participants received 30 vials, each containing 30 mL of the oil. The Governor EVOO was generously supplied by the oil producer OLIVE FABRICA from Greece, while *Bertolli oil* was purchased from the market. Participants were asked to include the olive oil (raw, not cooked) in their diet or drink it.

At baseline and after 6 months of olive oil intake, blood samples were collected to separate plasma and serum, followed by contrast-enhanced MRI and fMRI to measure BBB permeability and brain function, respectively. Participants were required to fast before each blood draw. Participants, investigators, and all research assistants were blinded to the treatment group, whereas one investigative team member (and one backup team member) were unblinded to randomization and prepared for each participant before each visit. All assays and MRI analyses were performed by investigators blinded to the type of olive oil each participant consumed. Participant compliance with olive oil consumption was confirmed by monthly interviews and by counting the empty vials delivered to the lab every month.

### 2.2. Inclusion/Exclusion Criteria

Twenty-six men and women aged between 55 and 75 years experiencing MCI according to one or more of the following criteria were included in the study, the Mini-Mental State Examination (MMSE) scores between 24–30 with memory complaint and/or Clinical Dementia Rating (CDR) score of 0.5. Only 25 participants completed the study. Participants were excluded if they were smokers or had clinically important medical or neuropsychiatric comorbidity. Patients with a cerebrovascular origin for dementia, such as a history of stroke, or those with major structural brain abnormalities such as tumors, alcohol and/or drug abuse, cerebrovascular pathology, other major psychiatric or neurological disorders, or renal problems were excluded from the study.

### 2.3. Neuropsychological Evaluation

For cognitive outcome, at the end of the olive oil consumption, participants were re-assessed for changes in cognition function measures between baseline and 6 months using MMSE, CDR, and Wechsler Memory Scale Fourth Edition (WMS-IV) scores. For WMS-IV logical memory (LM) scores, besides the overall score (total score of 27), we assessed immediate LM (LM I), delayed LM (LM II), and recognition LM (LM IIR) presented as % correct. WMS-IV visual reproduction (VR) was also assessed as follows; immediate VR (VR I), delayed VR (VR II), and recognition VR (VR IIR).

### 2.4. MRI Data Acquisition

The effect of olive oil consumption on BBB permeability and brain function was evaluated using contrast-enhanced MRI and fMRI, respectively. MRI data were acquired on a 7 Tesla MAGNETOM scanner (Siemens Healthcare, Erlangen, Germany) using a 32-channel head coil (Nova Medical, Wilmington, MA) at the AUMRIRC. The following types of MRI data were acquired: (i) Resting-state fMRI data were acquired using a multiband echo-planar imaging (EPI) sequence while participants did not perform any task and let their minds wander. The multiband EPI sequence used parameters: 51 slices, voxel size = 2.0 × 2.0 × 2.0 mm, TR/TE of 1000/22 ms, 70° flip angle, base/phase resolution 100/100, collected as slice-interleaved fashion, GRAPPA acceleration factor = 3, and multiband acceleration factor = 3; (ii) Functional MRI data were acquired while participants performed an n-back working memory task using the same sequence parameters as above; (iii) T1-weighted 3D structural MRI using the MPRAGE sequence before and after intravenous injection of gadobenate dimeglumine (Gd; Multihance^®^, Bracco Diagnostics; 0.05 mmol/kg), as an MRI contrast agent. The parameters used for these anatomical scans were: 256 slices, voxel size of 0.65 × 0.65 × 0.65 mm, TR/TE of 2200/2.8 ms, 7° flip angle, and base/phase resolution 384/100%.

### 2.5. N-Back Task

We used the 1-back task as a cognitive challenge. Stimuli were presented for 1500 ms with a 500 ms intertrial interval in blocks of 50 trials for 200 trials. Each block was separated by a 15 s rest period (total time = 7:25). The neural network actively involved in the n-back includes an extensive frontoparietal network, encompassing regions involved in attention and decision-making [21,22]. For both cognitive tasks, the number of correct responses, and reaction time (ms), were recorded by the E-Prime (https://pstnet.com/products/e-prime/ (accessed on 6 June 2022)) stimulus presentation software.

### 2.6. MRI Data Preprocessing

Neuroimaging data from 3 participants were excluded due to excessive motion or registration issues (2 from the ROO group and 1 from the EVOO group), leaving a total useable sample of *n* = 12 for the EVOO group and *n* = 10 for the ROO group.

Raw anatomical and functional neuroimaging data were converted to NIFTI format using dcm2niix (https://github.com/rordenlab/dcm2niix (accessed on 22 April 2022)) and organized in BIDS format. Results included in this study come from standard preprocessing performed using *fMRIPrep* 0.0.2 [23,24] (RRID: SCR_016216), which is based on *Nipype* 1.4.2 (RRID: SCR_002502) [25]. The anatomical and functional data preprocessing was performed as previously reported [26].

### 2.7. Task fMRI Activation Analysis

fMRI tasks were analyzed using FEAT (FMRI Expert Analysis Tool) Version 6.00, part of FSL (FMRIB’s Software Library, www.fmrib.ox.ac.uk/fsl) [21,27], as we previously reported [26].

Group z-statistic images were thresholded non-parametrically using clusters determined by z > 2.3 as well as a corrected cluster significance threshold of *p* < 0.05 using a within-subjects, repeated measures design (controlling for each individual’s average brain activation over their two imaging sessions to account for individual differences in global brain activity). Statistical images were generated for the task and the contrast between task and rest. A second level model was then constructed using individual subject task activation contrast maps to test the main effect of group, main effect of time (baseline vs. 6 months), and their interaction.

### 2.8. Resting-State fMRI Connectivity Analysis

Before estimating functional connectivity (FC), we reduced the dimensionality of the data by estimating the mean time series from pre-defined brain regions. The logic for doing so is that nearby voxels inside the brain are likely to engage in the same neural process. Therefore, it makes sense to parcellate the brain and use a single time series to represent a given macroscopic neural unit represented by a parcel or region of interest (ROI). For this purpose, we used the Power (pp272) brain parcellation atlas. We calculated the mean time series for each of the 272 ROIs covering the entire brain and estimated Pearson’s correlation coefficients between these mean time series to construct FC connectivity matrices across all ROIs in each participant. Using these matrices, pair-wise statistical tests were performed to compare the connectivity values between groups (ROO vs. EVOO) and between sessions (baseline vs. after 6 months of treatment). We report results for those connections that showed a significant interaction effect between groups and sessions.

### 2.9. Contrast-Enhanced MRI for BBB Permeability Analysis

Dynamic contrast-enhanced (DCE) MRI is the gold standard for assessing BBB permeability [28,29]. However, this requires dynamic scanning protocols, longer scanning sessions, and tracer injections during the scan. Given other sequences to run, including task and resting-state fMRI, the one-hour time window for the scan approved by IRB did not allow enough time for DCE-MRI. Therefore, we used the subtraction method instead for contrast-enhanced MRI. This method has been used previously to assess BBB permeability by Chassidim et al., where they showed that while dynamic methods provide better spatial accuracy, the subtraction method provides a slightly inferior, but acceptable alternative for interpretation purposes [30]. This method has been previously used by others, for example, in the assessment of BBB dysfunction as a biomarker for epileptogenesis [31]. We identified each participant’s hippocampus and parahippocampal gyrus (PHG) and estimated BBB permeability across all voxels within these regions. To do this, we used the T1 images obtained before and after injection of the contrast agent in the subtraction pipeline reported by Chassidim et al. [30]. This method provided a numerical value for BBB permeability for each hippocampal/PHG voxel in each subject at both baseline and 6-month scans. These were then subjected to statistical tests to determine whether there is a group × session interaction effect.

### 2.10. Measurements of Plasma Aβ_40_, Aβ_42_, Tau and p-tau181, and Serum NFL Using SIMOA

Venous blood samples were collected from all participants at baseline and end of olive oil consumption. Plasma and serum were separated, aliquoted, and stored at −80 °C within 30 min of collection. Plasma concentrations of Aβ_40_, Aβ_42_, tau, and p-tau181, and serum NFL were determined on the SIMOA HD-X analyzer (Quanterix; Billerica, MA, USA) using SIMOA Neurology 3-PlexA kit to assay plasma samples for Aβ_40_, Aβ_42_ and total tau, SIMOA pTau-181 (V2) kit to assay plasma p-tau 181, and SIMOA NF-Light kit to assay serum NFL [32].

### 2.11. Statistical Analysis

For derivatives of imaging data, a 2-way ANOVA was performed with group (EVOO vs. ROO) and time (baseline vs. 6-month) as the two main factors. For fMRI task activation, the individual subject contrast maps were input into the ANOVA while for resting state functional connectivity, we used connection strength of individual connections from each subject. For connections identified to have a significant interaction effect (*p* < 0.05, FDR corrected), paired *t*-tests were conducted for comparing every possible pair of groups. For BBB permeability analysis, we used the permeability calculated at each voxel within the hippocampus and PHG in the 2-way ANOVA and identified brain locations that showed a group × time interaction effect. Since an interaction effect in ANOVA does not guarantee significant pairwise difference between the groups, we also conducted paired *t*-tests (with permeability at each brain location as the sample) to determine statistical differences between permeability for each pair of groups.

For non-imaging data, we summarized all demographics and research variables using descriptive measures of central tendency and dispersion. For continuous variables measured at baseline and 6 months post olive oil consumption, sample means and standard deviations (SD) are reported. Changes in outcomes and biomarkers within treatment groups were tested using a one-sample *t*-test. Differences in baseline outcomes and biomarkers were tested using a two-sample *t*-test approach assuming unequal variances with a Satterthwaite approximation to the degrees of freedom. The normality assumption was examined for each test using histograms and normal probability plots. When the normality assumption appeared questionable or the influence of an extreme observation was a possible concern, appropriate non-parametric procedures of Wilcoxon Signed Rank and Wilcoxon Rank Sum were employed. Given the consistency across parametric and non-parametric procedures, all *p*-values reported are based upon parametric *t*-tests. All tests used a Type I error rate of 0.05. All tests were conducted using SAS 9.4 or R 4.1.2.

## 3. Results

### 3.1. Baseline Demographic and Clinical Characteristics

Subjects’ recruitment was conducted in the period from April 2019 to February 2020. Twenty-five participants completed the study. Thirteen participants were enrolled in the EVOO group and 12 participants in the ROO group. The baseline characteristics of the participants are presented in Table 1. The male participants represented 32% of the total participation. The study groups were well-matched without a statistically significant difference in their average age, body weight, education, MMSE, and CDR. Out of the 25 eligible participants, 5 individuals had CDR scores of 0, however, they showed questionable memory in their baseline MMSE and WMS-IV test scores and thus were included in the study.

### 3.2. Effect of ROO and EVOO on Functional Connectivity and BBB Permeability

**Figure 2:**
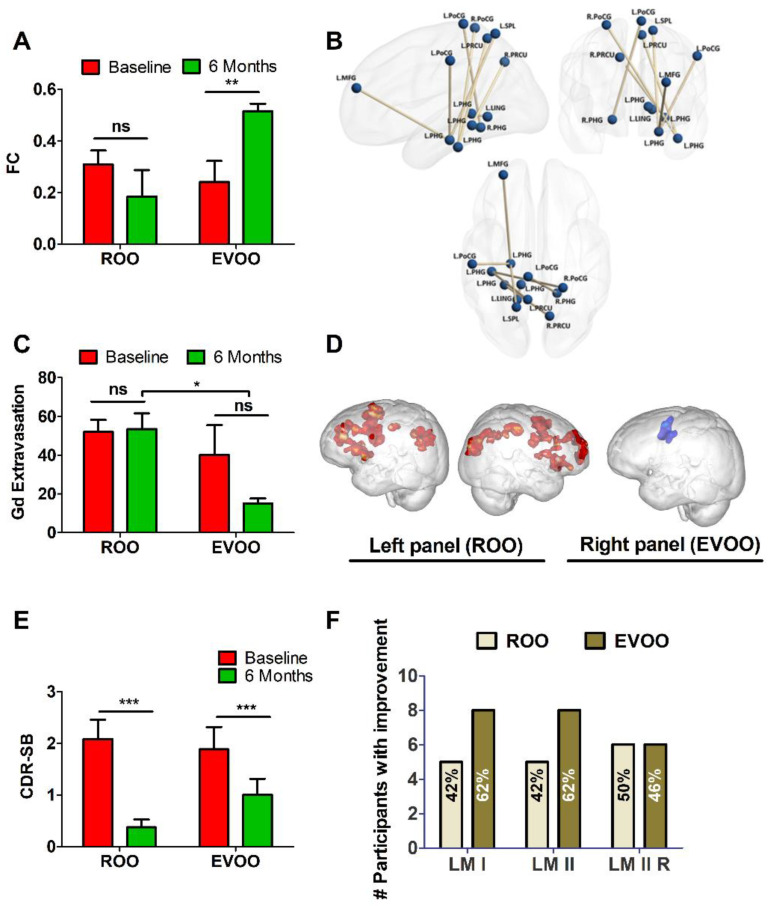
Findings from MRI screen. Effect of 6-month consumption of ROO and EVOO on (**A**) Functional connectivity (FC) for overall connections shown in Table 2. The baseline is shown in red and the 6-month timepoint is shown in green, with (**B**) Visualization of connections shown in Table 2. Abbreviation: Middle Frontal Gyrus (MFG), parahippocampal gyrus (PHG), precuneus (PRCU), Postcentral gyrus (PoCG), lingual gyrus (LING), superior parietal lobule (SPL). L: left and R: right (*n* = 12 in each group); and (**C**) BBB permeability assessed as overall Gd extravasation in the hippocampus and PHG shown in Table 2. (**D**) Comparison of network variant locations between task and rest states. The left panel is for ROO group 6-month > baseline task-rest differences, and the right panel is for EVOO group baseline > 6-month task-rest differences. Please see Tables S1 and S2 for a listing of coordinates from the local maxima within the cluster for ROO and EVOO groups, respectively. Effect of 6-month consumption of ROO and EVOO on (**E**) CDR-SB, and (**F**) the number of participants who demonstrated improved learning memory (immediate and delayed) assessed by the WMS-IV logical memory test. Data are presented as mean + SD; ns is not significant, * *p* < 0.05, ** *p* < 0.01, *** *p* < 0.001 is a significant difference between baseline and 6 months post olive oil consumption.

Among all possible connections between 272 ROIs, we identified eight functional connections that showed a group × session interaction effect (*p* < 0.05, FDR corrected for multiple comparisons). All the connections were between the PHG and regions such as bilateral precuneus, postcentral gyrus, lingual gyrus, middle frontal gyrus, and superior parietal lobule. Table 2 shows the pair-wise statistical comparison of the identified functional connections for both EVOO and ROO groups at both baseline and 6-month scans. Figure 2A displays a bar plot of the mean and standard deviation values of all connections taken together for each group and session. Figure 2B visualizes these functional connections in the brain using BrainNet Viewer software. As shown in Figure 2A, the groups had statistically similar connectivity values at baseline. However, the EVOO group’s connectivity increased significantly from baseline to 6 months post-consumption while the ROO group’s connectivity was not altered.

Corresponding results for BBB permeability, measured as Gd extravasation, are shown in Figure 2C and Table 2. Only the EVOO group demonstrated decreased BBB permeability in medial temporal lobe regions such as PHG and hippocampus compared to baseline. As shown in Table 2, the EVOO group demonstrated a significant reduction in BBB permeability in the individual medial temporal lobe regions after 6 months of consumption, which also showed a group × session interaction effect (*p* < 0.05, FDR corrected for multiple comparisons) suggesting the effect could be specific to EVOO compared to ROO (Table 2). Figure 2C demonstrates that the groups have statistically similar permeability values at baseline. While the EVOO group’s overall BBB permeability decreased post-EVOO, this reduction did not reach a significant difference which could be due to the small sample size. Yet, when compared to the ROO group, EVOO consumption demonstrated a significantly lower permeability compared to post-ROO suggesting improved BBB integrity. The ROO group’s BBB permeability was not affected.

### 3.3. Functional Neuroimaging and N-Back Task

None of the brain regions showed a significant interaction effect with respect to activation for the 1-back task. Therefore, we show brain regions that showed greater activation during the task as compared to the rest state for the following comparisons: baseline > 6-month and 6-month > baseline separately for both groups. Participants who received ROO demonstrated 6-month > baseline differences in a dispersed functional network inclusive of regions involved in perception and cognition (Figure 2D). Specifically, we observed greater activation in the cingulate gyrus (BA24/32), the left inferior and middle frontal gyri (BA9/44), the right inferior and middle frontal gyri (BA 9/47), bilateral inferior parietal lobe (BA40), and bilateral insula (BA13) (Figure 2D, and Table S1 in Supplementary material).

On the other hand, those who received EVOO showed less activation in areas related to motor responses (left middle and precentral gyri, BA6/9) (Figure 2D, and Table S2 in Supplementary material), for the baseline > 6-month comparison. No other significant differences were noted.

We calculated the % signal change during working memory performance. An ANOVA was conducted on the % signal change difference between 6 months and baseline. No significant differences emerged between EVOO and ROO groups (F (1, 20) = 0.719, *p* = 0.407). Therefore, task activation differences cannot be attributed specifically to the type of olive oil consumed. In the brain connectivity changes, we found that the specific effect of EVOO (i.e., the interaction effect) was limited to medial temporal lobe structures, which was confirmed in the BBB permeability results.

### 3.4. Effect of ROO and EVOO on Cognitive Measures

At baseline, there was no significant difference between the ROO and EVOO in participants’ body weight, MMSE, and CDR. In the WMS-IV logical memory test, the EVOO group subjects demonstrated lower LM overall score (12.5% vs. 16.3% for ROO group), immediate (45.8% vs. 59.3% for ROO group), and delayed recognition LM (80.3% vs. 83.7% for ROO group), yet this difference was not statistically different (*p* > 0.05; Table 3). However, a statistically significant difference between the 2 groups at baseline was observed in the delayed learning memory (LM-II, 31.5% vs. 55.2% for ROO group; *p* = 0.03) and visual memory recognition (2.2 vs. 3.8; *p* = 0.006) with EVOO group showing lower values as listed in Table 3.

ROO and EVOO daily intake did not alter the body weight. As summarized in Table 3, the ROO group body weight average values are 74.3 ± 12.4 (baseline) and 74.4 ± 13.1 (post), and the EVOO group average values are 85.1 ± 19.9 (baseline) and 85.7 ± 21.3 (post). These results suggest that 6 months of daily consumption of 30 mL olive oil have no effect on body weight.

Six months after olive oil daily consumption, the participants were reassessed for changes in cognitive function. While neither olive oil affected the MMSE, as shown in Table 3 and Table 4, a significant improvement in CDR rating was observed in the ROO group by (presented as difference (95% CI; *p*)) −0.292 (−0.455, −0.128; *p* = 0.0024) with 58% of subjects scoring 0 changing from 0.5. Similarly, the EVOO group demonstrated improvement in the CDR rating by −0.154 (−0.299, −0.0087; *p* = 0.0395) with 31% of subjects who scored 0.5 at baseline scoring 0 after 6 months of EVOO consumption. We also assessed the CDR-sum of boxes (CDR-SOB) rating. As shown in Figure 2E and Table 3, both ROO and EVOO reduced CDR-SOB significantly from 2.1 ± 1.29 to 0.38 ± 0.53 (*p* < 0.001), and 1.88 ± 1.56 to 1.0 ± 1.14 (*p* < 0.001), respectively, suggesting improved clinical outcomes.

For the WMS-IV logical memory test, as summarized in Table 4, a significant difference between baseline and after 6 months of daily consumption of EVOO was observed in the overall score increased by 2.034 points (0.0127, 4.08; *p* = 0.05) and LM-II by 13.45 (0.901, 25.99; *p* = 0.0377) suggesting improved memory. In addition, EVOO significantly improved visual reproduction recognition (VR IIR) by 1.46 (0.369, 2.554; *p* = 0.013), while ROO improved immediate visual reproduction (VR I) score by 3.33 (0.0089, 6.577; *p* = 0.045). While examining within each group demonstrated a significant difference, we cannot conclude the effects are different between the groups.

Furthermore, we assessed the effect of ROO and EVOO on participants’ improvement by calculating the % of participants who improved, did not change, or declined after 6 months of consumption. Participant’s improvement was defined as a change from baseline by >0%; no change as 0%; and a decline by <0% [33]. The results showed that those who consumed EVOO showed, while not statistically significant, a higher trend for improvement in the logical memory scores compared to the ROO group (Figure 2F). For the EVOO group, about 62% of participants showed improvement in LM I and LM II, and 46% in LM II recognition, while in the ROO group, 42% of the participants showed improvement in LM I and LM II, and 50% in LM II recognition (Figure 2B,F).

### 3.5. Effect of ROO and EVOO on Blood Biomarkers

At baseline, the analyzed blood biomarkers including Aβ_40_, Aβ_42_, Aβ_42_/Aβ_40_ ratio, p-tau 181, p-tau/tau ratio, and NFL were not significantly different between the ROO and EVOO groups (*p* > 0.05, Table 3), except in the levels of tau where the EVOO group showed significantly higher levels when compared to the ROO group (*p* = 0.035).

**Figure 3:**
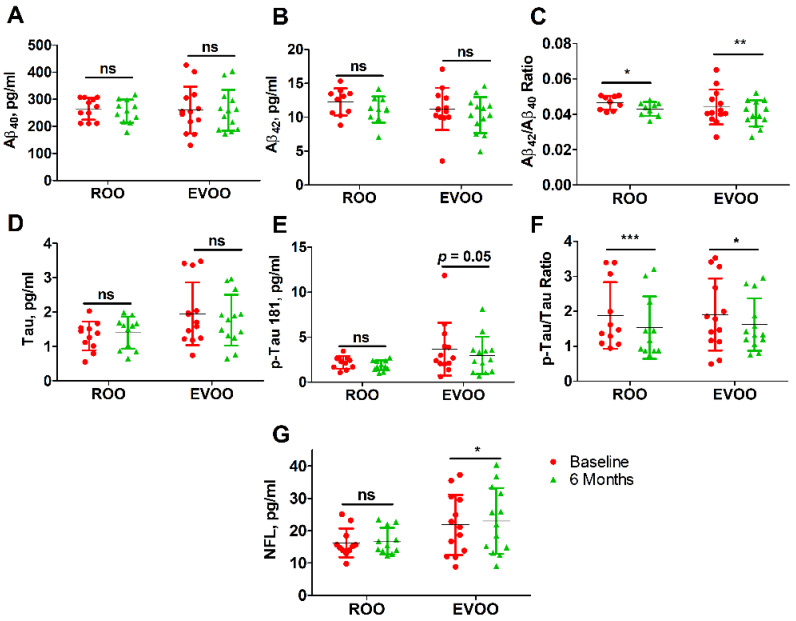
Effect of ROO and EVOO on changes in blood levels of AD biomarkers in MCI participants after 6 months of daily consumption. (**A**) Aβ_40_, (**B**) Aβ_42_, (**C**) Aβ_42_/Aβ_40_ ratio, (**D**) t-Tau, (**E**) p-Tau 181, (**F**) p-Tau/t-Tau ratio, and (**G**) NfL. Data are presented as mean ± SD; ns is not significant, * *p* < 0.05, ** *p* < 0.01, *** *p* < 0.001 between baseline and 6 months post olive oil consumption.

Compared to baseline, 6 months of daily consumption of either olive oil significantly altered Aβ_42_/Aβ_40_ and p-tau/tau ratios. As listed in Table 3 and Table 5 and shown in Figure 3, the ROO group demonstrated a significant reduction in Aβ_42_/Aβ_40_ ratio by 0.0034 (−0.0066, −0.00019; *p* = 0.041) and p-tau/t-tau ratio by 0.347 (−0.517, −0.179; *p* = 0.001). In the EVOO group, besides reducing Aβ_42_/Aβ_40_ by 0.0036 (−0.0061, −0.0012; *p* = 0.007) and p-tau/tau by 0.286 (−0.5561, −0.0175; *p* = 0.039), EVOO reduced p-tau 181 levels by 0.698 (−1.409, −0.011; *p* = 0.05). EVOO consumption was also associated with increased serum NFL levels by 1.206 pg/mL (0.050, 2.363; *p* = 0.042), however, this change was not significantly different between the olive oil groups, and thus we cannot conclude that the effects are different between ROO and EVOO groups.

## 4. Discussion

The consumption of olive oil is recognized for its beneficial effect on health. For example, in the most recent study that evaluated the effect of olive oil intake on total and cause-specific mortality demonstrated that the higher intake of olive oil was associated with lower total mortality and disease-specific mortality caused by cardiovascular, cancer, neurodegenerative and respiratory diseases [34]. In addition, in recent studies on the effect of EVOO intake on memory function in MCI subjects from Greece, the authors reported that the one-year intake of high- or med-phenolic EVOO improved cognitive function and altered AD blood biomarkers [35,36,37].

We recently reported the beneficial effect of EVOO in AD and CAA mouse models where EVOO addition to mice diet significantly reduced Aβ levels in the brain parenchyma and vasculature, reduced tau hyperphosphorylation, reduced neuroinflammation, and enhanced the BBB integrity and function which collectively improved memory function [16,17,18,19,20]. Based on these findings, the purpose of this pilot study was to translate our preclinical findings to human subjects with MCI and compare the effect of a high-phenolic content olive oil (EVOO) with refined olive oil that is purified from the phenolic content (ROO). This study was designed to measure two primary outcomes: 1) the effect of olive oil intake on BBB function using contrast-enhanced MRI, and 2) its effect on brain activity and network connectivity using fMRI after 6 months of daily consumption of EVOO and ROO, both are being investigated for the first time. Secondary outcomes measured were changes in cognitive function and blood AD-related biomarkers.

Findings from this randomized and double-blind pilot study in MCI participants demonstrated that the daily consumption of EVOO over 6 months could provide a beneficial effect on the brain by enhancing functional connectivity and reducing BBB permeability. Furthermore, MCI participants who consumed ROO also demonstrated increased activation in the fMRI task.

Neuroimaging studies in subjects with MCI and early AD demonstrated BBB breakdown in a number of brain regions before brain atrophy or dementia [37,38]. Studies have also shown alteration of blood vessel morphology where the vessel wall undergoes vascular endothelium changes leading to abnormal BBB permeability [39,40]. Deficits in BBB function can be imaged and quantified non-invasively using contrast-enhanced MRI [30,41]. Deficits in the BBB result in structural and functional consequences characterized by poor axonal integrity [42], and deficits in global and local network efficiency [43], respectively. Functional disruptions lead to deficits in cognition and behavior. In this study, the protective effect of olive oil on BBB was evaluated by contrast-enhanced-MRI. When compared to the baseline, findings demonstrated that EVOO significantly reduced the contrast extravasation in brain hippocampi and PHG after 6 months of daily consumption compared to ROO which did not alter the permeability of Gd (Figure 2C, Table 2). This improvement in BBB function is expected to lead to better axonal integrity, and higher local/global efficiency of functional brain networks, which can be seen from the fMRI findings where EVOO improved the functional connectivity between PHG and other cortical brain regions at the resting state. On the other hand, ROO participants demonstrated a greater activation following the n-back task, but there was no significant group × session interaction effect for task fMRI. Since working memory predominantly engages the dorsolateral prefrontal cortex, in retrospect, performing a task that predominantly engages the medial temporal lobe system might bring out the specific effect of EVOO in evoked response (similar to those observed in the resting state). These findings suggest that while the effects of ROO and EVOO were different, both olive oils could possess beneficial effects on the brain, which could imply the role of the phenolic content in differentiating EVOO from ROO and their effects; indeed, additional studies are necessary for confirmation.

Furthermore, we observed a significant improvement in memory in both olive oil groups, mainly in the CDR rating. At baseline, the participants’ average CDR-SOB values were 2.1 and 1.88 for ROO and EVOO groups, respectively, indicating a questionable impairment or MCI (CDR-SOB range 0.5–4) [44]. Pre-olive oil, 83% of ROO and 77% of EVOO participants demonstrated a CDR score of 0.5, which decreased to 25 and 46%, respectively, post-olive oil demonstrating a significant reduction in the number of participants with a CDR of 0.5 caused by olive oil consumption. Consistent with the CDR, in both groups, the CDR-SOB was significantly reduced, with the effect being more pronounced in the ROO group (ΔCDR-SOB of 1.72) compared to the EVOO (ΔCDR-SOB of 0.88), suggesting a significant memory improvement. Indeed, this is a noticeable effect that necessitates replication studies with increased subject numbers to confirm these findings.

In addition, the EVOO group demonstrated a significant improvement in WMS-IV LM overall score and delayed LM; however, there was no significant difference between the groups. Yet, it is important to note that there was no significant decline in LM in the ROO group, and while examining the effect within the EVOO group demonstrated a significant effect, we cannot conclude the effects are different between the groups. Therefore, additional investigation is required to confirm these results.

We next analyzed core AD blood biomarkers (Aβ_40_, Aβ_42_, t-tau, p-tau 181, and NFL) in the study participants. Previous longitudinal studies had shown that low plasma Aβ_42_/Aβ_40_ ratios are associated with an increased risk of MCI and AD [45]. While Aβ_40_, Aβ_42_, and t-tau were not significantly affected by either olive oil following 6 months of daily consumption, our findings showed that both ROO and EVOO significantly reduced Aβ_42_/Aβ_40_ and p-tau/t-tau ratios. Brain and blood Aβ levels are determined by Aβ clearance and production. Previous results from our preclinical studies have demonstrated that EVOO reduces brain Aβ levels by reducing its production and increasing its clearance by degradation and across the BBB in CAA/AD mouse models [16,17,18]. In the blood, Aβ clearance is also mediated by hepatic and renal clearance, and thus inducing these processes could also alter blood Aβ levels [46,47,48]. While further studies are necessary to confirm these results, reduced Aβ ratios could be explained by reduced production and increased peripheral clearance of Aβ. Similarly, ROO and EVOO significantly reduced the p-tau/t-tau ratio, an effect that could be influenced by the mild reduction in blood p-tau 181 (ROO, not significant; EVOO, *p* = 0.05), which is consistent with our preclinical observations in TgSwDI and 3xTg mouse models where EVOO reduced tau neuropathology [18,19,20]. The results also suggest reduced p-tau/t-tau ratio could be affected by the reduced Aβ levels and thus reduced tau phosphorylation [49,50,51]. Besides, we also determined the effect of olive oil consumption on serum NFL levels. While EVOO showed a mild but significant increase in NFL levels (*p* = 0.042), there was no significant difference between the two groups. Indeed, additional work is required to confirm and explain this finding, however, blood NFL is not specific to one disease and its levels can also be influenced by physiological and pathological conditions including age, body mass index, medications, diabetes, and hypertension to list a few [52].

This study has several limitations, notably, the small sample size, and the absence of control groups including MCI subjects who did not receive olive oil and age-matched subjects with normal cognitive function to compare brain and BBB function and blood AD biomarkers. Another limitation is the short treatment time (6 months); longer studies are necessary to better assess the long-term ingestion of olive oil on the brain and cognitive function, which could allow better differentiation between EVOO and ROO. While a significant difference was observed by one olive oil but not the other, group × session interaction for some assessed outcomes was not significant which makes it difficult to conclude whether the observed effect is EVOO or ROO specific. Indeed, findings from this proof-of-concept pilot study support conducting larger and well-controlled clinical trials to assess and compare olive oil’s protective effects against AD and its potential role in preventing MCI and its conversion to AD and related dementias. Finally, as mentioned before, DCE-MRI is the gold standard for assessing BBB permeability [28,29]. Given the one-hour time window for the scan approved by IRB and other MRI scans we had to do including anatomical, task and resting fMRI, we had time only for pre and post contrast T1 scans and not DCE-MRI. Therefore, we ended up using the subtraction method for assessing BBB permeability. Given the results of the current study, it makes a strong case for dedicating more of the MRI time to DCE-MRI for BBB permeability assessment in similar future clinical trials.

## 5. Conclusions

In conclusion, findings from this pilot study showed that EVOO consumption resulted in improvements in BBB function as demonstrated by the increase in its restrictive ability to permeate Gd, a contrast molecule that has limited access to the brain, and enhanced brain function and memory in individuals with MCI. In addition, findings showed the beneficial effects not only of EVOO but with ROO, which suggests that besides the health benefits of EVOO-phenolic compounds, the monounsaturated fats (such as oleic acid as the major fat in olive oil), which are present in both EVOO and ROO could contribute to this effect.

## Figures and Tables

**Table 1:** Baseline demographic and clinical characteristics of study participants.

Variable	ROO (*n* = 12)	EVOO (*n* = 13)	*p* Value
Male	3 (25%)	5 (38%)	
Female	9 (75%)	8 (62%)	0.47
Age (years)	65.5 ± 4.4	67.5 ± 5.5	0.31
Bodyweight (kg)	74.± 3 ± 12.4	85.1 ± 19.9	0.11
Education (years)	15.8 ± 2.7	14.2 ± 3.7	0.25
MMSE	27.83 ± 2.17	27.15 ± 2.64	0.49
CDR	0.42 ± 0.19	0.38 ± 0.22	0.87

For males and females, data are presented as participants number (% of total number of partici-pants). The remaining data are presented as mean (SD).

**Table 2:** Pair-wise statistical comparison of identified functional connections and BBB permeability for ROO and EVOO groups. Bold values indicate significantly different.

Functional Connection	Pair-Wise *p*-Values				
	EVOO2 > EVOO1	ROO2 vs. ROO1	EVOO1 vs. ROO2	EVOO1 vs. ROO1	EVOO2 > ROO2
L Precuneus ↔ L Parahippocampal Gyrus	**0.02**	0.46	0.83	0.66	**0.01**
R Precuneus ↔ L Parahippocampal Gyrus	**0.02**	0.41	0.78	0.50	**0.05**
L Postcentral Gyrus ↔ R Parahippocampal Gyrus	**0.03**	0.93	0.80	0.77	**0.04**
L Postcentral Gyrus ↔ L Parahippocampal Gyrus	**0.05**	0.76	0.98	0.80	**0.04**
R Postcentral Gyrus ↔ L Parahippocampal Gyrus	**0.05**	0.31	0.50	0.91	**0.01**
L Lingual Gyrus ↔ L Parahippocampal Gyrus	**0.04**	0.22	0.84	0.34	**0.05**
L Middle Frontal Gyrus ↔ L Parahippocampal Gyrus	**0.05**	0.65	0.36	0.52	**0.01**
L Superior Parietal Lobule ↔ L Parahippocampal Gyrus	**0.04**	0.06	0.52	0.35	**0.01**
**BBB permeability**					
L Parahippocampal Gyrus	**0.00**	0.60	0.69	0.27	**0.00**
R Parahippocampal Gyrus	**0.01**	0.85	0.34	0.40	**0.04**
L Hippocampus	**0.03**	0.87	0.15	0.21	**0.05**
R Hippocampus	**0.04**	0.99	0.70	0.71	**0.03**

The baseline is presented as (1) and the 6-month is presented as (2). For example, EVOO1 corresponds to the EVOO group at baseline (session 1) and EVOO2 is after 6 months (session 2). Significant *p*-values (*p* ≤ 0.05 FDR corrected) are shown in boldface.

**Table 3:** Changes in cognitive outcomes and blood biomarkers.

Variable	ROO (*n* = 12)		EVOO (*n* = 13)	
	Baseline (SD)	6 Months (SD)	Baseline (SD)	6 Months (SD)
Bodyweight (kg)	74.3 (12.4)	74.4 (13.1)	85.1 (19.9)	85.7 (21.3)
MMSE	27.83 (2.17)	28.83 (1.47)	27.15 (2.64)	26.92 (2.78)
CDR	0.42 (0.19)	0.13 (0.23) *	0.38 (0.22)	0.23 (0.26) *
% with CDR 0.5	83%	25%	77%	46%
CDR-SOB	2.1 (1.29)	0.38 (0.53) ***	1.88 (1.56)	1.00 (1.14) ***
WMS-IV Logical Memory				
LM overall score	16.3 (4.9)	15.9 (4.4)	12.5 (4.5)	14.5 (5.3) *
%Correct				
LM I	59.3 (18.9)	56.3 (20.8)	45.8 (19.6)	52.4 (20.4)
LM II	55.2 (27.0)	50.4 (17.7)	31.5 (24.1) #	44.9 (31.2) *
LM II Recognition	83.7 (12.3)	88.1 (11.7)	80.3 (16.2)	82.5 (15.6)
VR I	37.9 (5.2)	41.3 (3.5) *	38.9 (6.5)	41.2 (2.7)
VR II	41.3 (2.9)	40.2 (4.6)	42.8 (0.8)	42.4 (2.2)
VR II Recognition	3.8 (1.2)	3.8 (1.4)	2.2 (1.5) #	3.7 (1.6) *
Blood biomarkers (pg/mL)				
Aβ40	265 (40)	256 (43)	260 (86)	259 (75)
Aβ42	12.2 (2.0)	11.1 (1.9)	11.2 (3.1)	10.3 (2.7)
Aβ42/Aβ40	0.047 (0.004)	0.044 (0.005) *	0.044 (0.009)	0.040 (0.007) **
Tau	1.30 (0.42)	1.40 (0.46)	1.95 (0.91) #	1.76 (0.74)
p-Tau181	2.19 (0.70)	1.85 (0.56)	3.67 (2.94)	2.97 (2.08) *
p-Tau/t-Tau	1.88 (0.95)	1.53 (0.89) **	1.91 (1.03)	1.62 (0.75) *
NFL	16.2 (4.5)	16.8 (4.1)	21.8 (9.3)	23.0 (10.2) *

Data are presented as mean (SD). * *p* < 0.05, ** *p* < 0.01, *** *p* < 0.001, # *p* < 0.05. * Compared to baseline. # Compared to ROO at baseline.

**Table 4:** Changes in MMSE, CDR, and WMS-IV scores after 6 months of olive oil consumption.

	ROO (*n* = 12)		EVOO (*n* = 13)		
	Mean ^a^ (SD)	*p*	Mean ^a^ (SD)	*p*	95% CI for Difference of Differences
MMSE	1.00 (2.73)	0.23	−0.23 (1.54)	0.60	(−0.583, 3.044)
CDR	−0.291 (0.257)	**0.0024**	−0.154 (0.240)	**0.039**	(−0.344, 0.068)
WMS-IV					
Overall score	−0.323 (5.068)	0.83	2.034 (4.347)	**0.05**	(−5.897, 1.182)
LM-I	−3.036 (20.9)	0.63	6.615 (14.9)	0.13	(−24.573, 5.272)
LM-II	−4.786 (29.17)	0.58	13.451 (20.77)	**0.038**	(−39.056, 2.582)
LM-IIR	4.375 (9.80)	0.15	2.244 (18.57)	0.67	(−10.321, 14.591)
VR I	3.333 (5.10)	**0.04**	2.308 (4.35)	0.08	(−2.887, 4.938)
VR II	−1.167 (4.43)	0.38	−0.385 (2.43)	0.58	(−3.706, 2.142)
VR IIR	0.0 (1.13)	1.00	1.462 (1.81)	**0.013**	(−2.721, −0.202)

^a^ Mean is for mean difference (i.e., 6 months−baseline). Bold numbers indicate a statistically significant difference.

**Table 5:** Changes in AD blood biomarkers after 6 months of olive oil consumption.

	ROO (*n* = 12)		EVOO (*n* = 13)		
	Mean ^a^ (SD)	*p*	Mean ^a^ (SD)	*p*	95% CI for Differences of Differences
Aβ_40_	−8.835 (59.6)	0.63	−1.150 (56.8)	0.94	(−57.038, 41.669)
Aβ_42_	−1.29 (1.85)	0.07	−0.901 (2.15)	0.158	(−2.235, 1.456)
Tau	0.098 (0.42)	0.46	−0.180 (0.69)	0.368	(−0.220, 0.770)
p-Tau 181	−0.344 (0.66)	0.12	−0.698 (1.18)	**0.05**	(−0.476, 1.184)
Aβ_42_/Aβ_40_ ratio	−0.0034 (0.004)	**0.041**	−0.0036 (0.004)	**0.007**	(−0.003, 0.004)
p-Tau/Tau ratio	−0.347 (0.251)	**0.001**	−0.286 (0.446)	**0.039**	(−0.376, 0.253)
NFL	0.553 (2.25)	0.43	1.206 (1.91)	**0.042**	(−2.414, 1.109)

^a^ Mean is for mean difference (i.e., 6 months−Baseline). Bold numbers indicate a statistically significant difference.

## Data Availability

Data described in the manuscript will be made available upon request pending application to the corresponding author and stipulation that the data will not be used for commercial purposes.

## Supplementary Materials

The following supporting information can be downloaded at: https://www.mdpi.com/article/10.3390/nu14235102/s1, Table S1: ROO 6 months > baseline task-rest differences; Table S2: EVOO group baseline > 6-month task-rest differences; EVOO Certificate of Analysis.
