# Supplementary material for: Extra-Virgin Olive Oil Enhances the Blood–Brain Barrier Function in Mild Cognitive Impairment: A Randomized Controlled Trial
Source: Nutrients. 2022 Dec 1;14(23):5102. doi: 10.3390/nu14235102 (PMC9736478; doi:10.3390/nu14235102)
Supplement: Supplementary file 1 [file nutrients-14-05102-s001.zip › nutrients-2068114Supplementary Material.pdf]

Extra-virgin olive oil enhances the blood-brain barrier function in mild cognitive impairment: a randomized controlled trial

Amal Kaddoumi[1,2]*, Thomas S. Denney Jr.[2,3], Gopikrishna Deshpande[2-9], Jennifer L. Robinson[2,3,4], Ronald J. Beyers[3], David T. Redden[10], Domenico Pratico[11], Tassos C. Kyriakides[12], Bonian Lu[3], Anna N. Kirby[13], Darren T. Beck[14], Nancy D. Merner[15]

[1] Department of Drug Discovery and Development, Harrison College of Pharmacy, Auburn University. Auburn, AL, USA

[2] Center for Neuroscience, Auburn University, Auburn, AL, USA.

[3] Auburn University MRI Research Center, Department of Electrical & Computer Engineering, Auburn University, Auburn, AL, USA

[4] Department of Psychological Sciences, Auburn University, Auburn, AL, USA.

[5] Alabama Advanced Imaging Consortium, Birmingham, AL, USA.

[6] Key Laboratory for Learning and Cognition, School of Psychology, Capital Normal University, Beijing, China.

[7] Department of Psychiatry, National Institute of Mental Health and Neurosciences, Bangalore, India.

[8] Centre for Brain Research, Indian Institute of Science, Bangalore, India.

[9] Department of Heritage Science and Technology, Indian Institute of Technology, Hyderabad, India.

[10] Biomedical Affairs and Research, Edward Via College of Osteopathic Medicine, Auburn University. Auburn, AL, USA

[11] Alzheimer's Center at Temple, Lewis Katz School of Medicine Temple University, Philadelphia, PA, USA

[12] Yale School of Public Health, New Haven, CT, USA

[13] Department of Cell Biology and Physiology and Preventive Medicine and Public Health, Edward Via College of Osteopathic Medicine, Auburn University. Auburn, AL, USA

[14] Department of Cell Biology and Physiology, Via College of Osteopathic Medicine, Auburn University. Auburn, AL, USA

[15] Department of Pathobiology, College of Veterinary Medicine, Auburn University. Auburn, AL, USA

* Correspondence

Amal Kaddoumi, Department of Drug Discovery & Development, Harrison College of Pharmacy, Auburn University, 720 S. Donahue Dr., Auburn, AL 36849.

Email: kaddoumi@auburn.edu

Table S1. ROO 6 months > baseline task-rest differences.

z	x	y	z	Lobe	Region	BA
3.09	-50.77	10.85	24.92	Frontal	Left Inferior Frontal Gyrus	9
3.02	-43.23	1.42	22.54			
2.98	-47.01	4.89	28.17			
3.07	-54.55	10.99	23.09			44
2.94	-45.11	1.66	18.93			
3.30	0.23	1.07	51.74		Left Medial Frontal Gyrus	6
3.34	-31.93	7.04	51.93		Left Middle Frontal Gyrus	6
3.30	-28.10	13.05	46.91			
3.01	-26.23	14.35	56.06			
2.99	-26.23	7.34	46.55			
2.58	-24.36	5.20	50.05			
2.55	-24.35	1.75	44.39			
3.20	-37.46	27.79	26.10			9
3.73	-39.30	39.41	23.23			10
3.14	-35.50	39.73	17.84			
2.64	-26.02	39.88	14.29			
2.51	-31.72	43.14	23.52			
2.49	-27.93	43.11	23.54			
3.37	0.24	4.87	51.99		Left Superior Frontal Gyrus	6
3.13	34.58	33.04	-5.69		Right Inferior Frontal Gyrus	47
2.37	30.77	30.83	-0.42			
2.87	40.27	43.90	2.30			
3.46	2.19	14.79	45.41		Right Medial Frontal Gyrus	6
3.41	36.43	46.10	27.75		Right Middle Frontal Gyrus	9
3.06	36.44	46.33	24.14			10
2.98	36.42	40.65	23.77			
2.98	40.24	46.53	20.56			
3.05	40.26	47.00	13.35			
3.04	40.27	50.79	13.60			
3.02	34.59	43.84	4.07			
2.77	30.79	43.52	9.45			
2.76	28.92	49.34	8.01			
2.90	32.59	34.41	32.39		Right Superior Frontal Gyrus	9
3.29	34.57	50.26	22.58			10
2.95	-3.48	9.73	35.99	Limbic	Left Cingulate Gyrus	24
3.38	-1.60	9.49	39.61			32
2.87	-11.06	9.68	37.75			
3.18	26.53	-76.93	17.87	Occipital	Right Cuneus	18
4.01	30.29	-75.76	28.83			19
3.60	30.27	-81.33	26.66			
4.16	30.30	-75.53	25.23		Right Middle Occipital Gyrus	19
3.35	-35.91	-58.35	33.17	Parietal	Left Angular Gyrus	39
3.06	-32.11	-52.81	35.37		Left Inferior Parietal Lobule	40
3.04	-56.65	-35.53	36.34			
3.17	-43.40	-37.31	32.69		Left Supramarginal Gyrus	40
3.11	-37.77	-48.85	33.78			
3.23	49.36	-30.89	39.10		Right Inferior Parietal Lobule	40
3.19	51.20	-40.73	43.90			
3.10	47.44	-35.01	44.25			
3.06	41.74	-38.87	45.78			
3.22	49.36	-29.23	42.83		Right Postcentral Gyrus	2
2.85	28.41	-64.95	38.57		Right Precuneus	7
3.35	32.19	-72.22	32.69			19
3.12	-33.69	8.30	3.14	Sub-lobar	Left Claustrum	
2.92	-27.94	25.42	2.48			
3.59	-31.76	19.54	5.69		Left Insula	13
3.15	-39.34	12.37	-0.25			
3.67	28.84	23.15	0.87		Right Claustrum	
3.64	28.84	17.81	-4.90		Right Insula	13
3.54	36.37	15.15	5.83			
2.65	40.16	11.67	0.20			
3.54	-32.12	-60.16	31.27	Temporal	Left Middle Temporal Gyrus	39

Table S2. EVOO group baseline > 6-month task-rest differences.

z	x	y	z	Lobe	Region	BA
3.12	-37.58	9.34	46.61	Frontal	Left Middle Frontal Gyrus	6
2.83	-33.81	3.85	42.66			
3.15	-43.23	8.30	33.85			9
2.83	-47.00	12.13	34.07			
2.98	-37.58	2.45	35.31		Left Precentral Gyrus	6
2.84	-33.79	0.75	31.60			

C2122-00408

World Olive Center for Health

76 Imittou St. 5th floor
11634, Pagkrati, Athens
Tel: 2107010131
info@worldolivecenter.com

Athens: **30/11/2021**
Cert. Num: **C2122-00408**

CERTIFICATE OF ANALYSIS

**Brand Name:**	The Governor Limited Edition	**Analysis Date:**	09/11/2021
**Owner:**	G DAFNIS AND S DAFNIS SA		
**Variety:**	LIANOLIA		
**Origin:**	AGIOS MATTHAIOS KERKYRA GREECE		
**Harvesting Period:**	October 2021		
**Oil Press:**	G DAFNIS AND S DAFNIS SA		

Chemical Analysis

Oleocanthal	621	mg/Kg
Oleacein	344	mg/Kg
Oleocanthal+Oleacein (index D1)	965	mg/Kg
Ligstroside aglycon (monoaldehyde form)	44	mg/Kg
Oleuropein aglycon (monoaldehyde form)	41	mg/Kg
Ligstroside aglycon (dialdehyde form)*	97	mg/Kg
Oleuropein aglycon (dialdehyde form)**	34	mg/Kg
Free Tyrosol	20	mg/Kg
Total tyrosol derivatives	782	mg/Kg
Totalhydroxytyrosol derivatives	419	mg/Kg
Total polyphenols analyzed	1.201	mg/Kg

Comments:

The chemical analysis was performed according to the method that has been submitted to EFET and published in J Agric Food Chem, 2012, 60,11696, J Agric Food Chem, 2014,62, 600-607 & Molecules, 2020, 25, 2449. *Oleomissional+Oleuropeindial **Ligstrodial+Oleokoronal

Magiatis Prokopios

PROKOPIOS MAGIATIS
ASSOCIATE PROFESSOR
UNIVERSITY OF ATHENS
FACULTY OF PHARMACY
DEPARTMENT OF PHARMACOGNOSY
AND NATURAL PRODUCTS CHEMISTRY
